# High-Energy Radiation Effects on Silicon NPN Bipolar Transistor Electrical Performance: A Study with 1 MeV Proton Irradiation

**DOI:** 10.3390/ma16216977

**Published:** 2023-10-31

**Authors:** Haddou EL Ghazi, Redouane En-nadir, Anouar Jorio, Mohamed A. Basyooni-M. Kabatas

**Affiliations:** 1 2SMPI Group, ENSAM Laboratory, Hassan II University, Nile 150, Casablanca 20670, Morocco; hadghazi@gmail.com; 2 LPS, Faculty of Sciences, Mohamed Ben Abdellah University, Fes 30000, Morocco; a_jorio@hotmail.com; 3 Department of Precision and Microsystems Engineering, Delft University of Technology, Mekelweg 2, 2628 CD Delft, The Netherlands; 4 Department of Nanotechnology and Advanced Materials, Graduate School of Applied and Natural Science, Selçuk University, Konya 42030, Turkey

**Keywords:** silicon transistors, NPN-BJTs, emitter-base, proton irradiation, electric performance

## Abstract

This study investigates the degradation of the silicon NPN transistor’s emitter-base junction, specifically the 2N2219A model, under both forward and reverse polarization. We examine the current–voltage characteristics under the influence of 1 MeV proton irradiation at various fluencies, which are $5.3\times 10^{8},5.3{\times 10}^{10},5\times 10^{11},5\times 10^{12}$, and $5{\times 10}^{13}$ protons/cm², all conducted at 307 K. The experimental findings elucidate a pronounced dependency of diode parameters, including the reverse saturation current, series resistance, and the non-idealist factor, on the incident proton flow. This observation underscores that proton-induced degradation is primarily driven by displacement damage, while recorded degradation is predominantly attributed to the generation of defects and interfacial traps within the transistor resulting from exposure to high-energy radiation. Our findings indicate that the effects of irradiation align more closely with the compensation phenomenon in doping rather than its reinforcement.

## 1. Introduction

In today’s world, where nuclear technology, satellites, and aeronautics are playing increasingly vital roles, the demand for semiconductor devices capable of withstanding radiation-rich environments has grown significantly. This demand has ignited a substantial body of research dedicated to addressing this critical requirement.

Silicon-based bipolar junction transistors (BJTs) have emerged as prominent candidates for such applications, primarily due to their versatile functionality in various electronic circuits [1,2,3]. A particular emphasis is placed on scrutinizing the emitter-base junction of silicon NPN transistors, as these transistors constitute integral components of modern electronics [4,5].

Understanding the behavior of silicon-based BJTs, especially their emitter-base junction, under irradiation conditions, is paramount. BJTs play a pivotal role in signal amplification, switching applications, and numerous electronic systems. Their reliable performance in radiation-rich environments is crucial for the successful operation of nuclear technology, satellites, aeronautics, and other critical applications. For example, the impact of radiation on FET-based devices can result in a range of effects, including changes in electrical characteristics, performance degradation, and the introduction of defects or traps within the device, ultimately influencing its functionality and reliability [6,7,8]. This study presents an opportunity to innovate radiation monitoring devices, particularly radiation-sensitive metal-oxide-silicon field-effect transistors (RADFETs) and dosimeter devices based on PNP structures. RADFETs have been utilized effectively in space missions and laboratories to measure radiation doses. They operate by generating a continuous electrical output, which is electronically converted to accurately assess accumulated dose. This remote monitoring and display capability makes them versatile [9].

To ensure the reliability and functionality of these electronic components in demanding conditions, it has become imperative to investigate the effect of irradiation on the electrical performance of these devices. To achieve this effectively, we must undertake comprehensive studies employing particles capable of inducing defects in semiconductor materials. Among these various particles, protons stand out due to their distinctive advantage—they generate defects at a significantly higher rate compared to other particles such as neutrons and electrons [10].

Extensive investigations have been conducted to explore irradiation-induced damage and establish effective procedures for radiation hardening of metal-oxide-silicon (MOS) and bipolar silicon (Si) devices, which are amenable to seamless integration [11,12,13,14,15]. In this context, Ohyama et al. have studied the degradation in the electrical characteristics of silicon junctions, both n + p and p + n, in response to the influence of 1 MeV neutron and 1–2 MeV electron irradiation [16]. Hefner et al. have investigated the influence of neutrons on the characteristics of the insulated gate bipolar transistor (IGBT) [17]. Omotoso et al. have investigated the impact of high-energy electron irradiation on the Schottky barrier height and the Richardson constant of Ni/4H-SiC Schottky diodes [18].

Recently, Pearton and co-workers have demonstrated the impact of ionizing radiation damage effects on GaN devices [19]. The same team has demonstrated that the proton and electron irradiation in HEMTs induces positive threshold voltage shifts by trapping electrons at defects, reducing carrier mobility, and degrading drain current and transconductance [20]. Schwarz et al. explored the effects of gamma irradiation on electronic carrier transport in AlGaN/GaN high electron mobility transistors (HEMTs) [21].

Previous investigations have highlighted the deterioration of electric properties in silicon junctions subjected to irradiation. In our previous research, we have demonstrated a significant impact on the optical properties of silicon junctions when exposed to irradiation. Specifically, our findings indicate that, under forward bias conditions and an irradiation fluence of $5\times 10^{12}\;$protons/cm², the electroluminescence intensity experiences a pronounced reduction of up to 96% [22,23,24].

This study serves as the foundation for an experimental comprehensive investigation into the impacts of 1 MeV proton irradiation on the electric characteristics of silicon emitter-base bipolar junction transistors (BJTs), with a primary focus on the emitter-base junction within silicon NPN transistors, particularly the commercially available 2N2219A model by ST Microelectronic.

## 2. Experimental Setup and Materials

**Figure 1:**
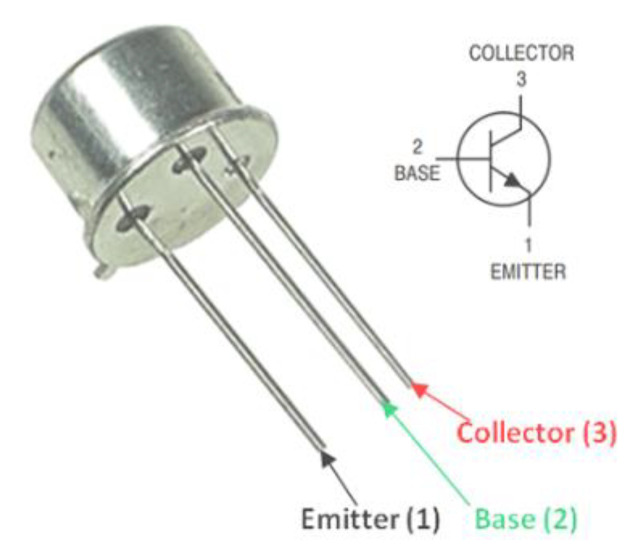
The official design of the commercialized 2N2219-NPN transistor by STMicroelectronics Inc. features the schematic electronic symbol. Adapted from [25].

For this study, silicon emitter-base transistors bipolar (NPN) of the 2N2219A model, commercially manufactured by ST Microelectronics (Geneva, Switzerland), were selected as test samples. These transistors featured fixed emitter and base areas measuring 0.01 mm². Sample preparation included metalizing the top surface of the transistors with a uniform 1.6 µm layer of aluminum, while the higher face underwent SiO₂ passivation and the back face was coated with 0.3 µm of gold. Figure 1 shows the commercialized 2N2219A NPN transistor investigated in this study. The 2N2219A is a silicon Planar Epitaxial NPN transistor encased in a metal body, conforming to the Jedec TO-39 standard (2N2219A) and the TO-18 standard (2N2222A) [25]. This transistor is specifically engineered for high-speed switching applications and can handle collector currents of up to 500 mA. In addition, it exhibits a favorable current gain across a broad spectrum of collector current values, along with minimal leakage currents and low saturation voltage. A datasheet containing the technical specifications for the 2N2219A NPN transistor can be found on the STMicroelectronics website [25].

The primary focus of the investigation centered on the behavior of the emitter-base (E-B) junction, with the collector terminal being left in a floating state. To characterize the current–voltage (I–V) characteristics, a parametric analyzer under computer control via an IEEE bus interface was employed. Irradiation was performed using a Faraday cup designed for the precise measurement of proton fluences. To ensure rigorous control over experimental parameters, a proton beam source was employed, maintaining a current not exceeding 3 nA. Stringent control was exercised over various parameters to facilitate an accurate comparison of degradation behavior, including proton beam flux and energy, constant irradiation temperature at 307 K, and controlled elapsed time between irradiation and measurement. Current–voltage measurements were conducted precisely 5 min post-irradiation cessation to assess immediate proton-induced effects. Measurement precision was maintained at a standard deviation of 3% by employing a total of 15 diodes in the experimental setup. All experimental measurements were executed within the electrical engineering and nuclear laboratories situated in the physics departments of Sherbrook and Montreal universities in Canada.

## 3. Results and Discussion

To elucidate the current–voltage characteristics of our specimens, we initiate our analysis by delineating the constituent current components contributing to the overall junction current. For the case of forward polarization, we consider two principal components: the bulk diffusion current originating from the neutral region, and the bulk generation-recombination current, which arises within the depletion region. Accounting for the resistivity attributed to the distinct regions comprising the junction, namely the emitter, base, and ohmic contact, the total current can be expressed as follows [26]:

(1) $$I=I_{s}\left[\exp\left(\frac{q\left(V-R_{s}I\right)}{nk_{B}T}\;\right)\right]-1,$$

where $R_{s}$ is the series resistance, $I_{s}\;$is the reverse saturation current, n is the non-ideality factor, q is the electron charge, T is the absolute temperature, and $k_{B}\;$is the Boltzmann’s constant. The measure of n depends on which current component is dominated in a specific operating regime.

For reverse polarization, the primary contribution to the junction current stems from the generation from deep centers. In our model, we neglect the tunneling component. This current dependency is contingent upon both temperature and the applied voltage, and can be described as follows [26]:

(2) $$I_{R}\propto \sqrt{T}\sqrt{V_{R}}\exp\left(-\frac{E_{\alpha }}{k_{B}T}\right)\exp\left(CV_{R}\right),$$

where $V_{R}$ is the reverse applied voltage, $E_{\alpha }\;$is the activation energy, and C is a constant that depends on the semiconductor material.

**Figure 2:**
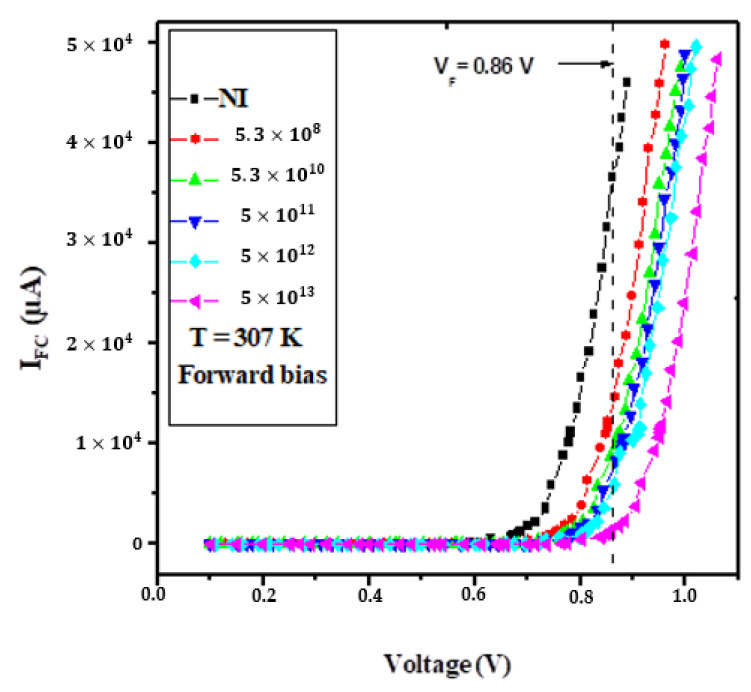
The forward bias I–V characteristics of bulk silicon (NPN) emitter-base at 307 K are presented, incorporating the impact of 1 MeV proton irradiation.

Figure 2 depicts a comprehensive overview of the alterations in the current–voltage (I–V) characteristics of silicon-based NPN (emitter-base) junction subjected to forward polarization, while also taking into account the impact of irradiation at an operating temperature of 307 K. A prominent and intriguing observation within these characteristics is the discernible increase in the threshold voltage as the irradiation fluence escalates. This trend is of particular significance as it suggests that the junction becomes less conducive to forward bias operation as it encounters higher levels of irradiation.

Moreover, when maintaining a constant applied voltage across the NPN junction, the current demonstrates a consistent and notable decline in response to increasing irradiation fluences. The observed reduction in current flow due to the influence of irradiation carries significant implications for the performance and behavior of silicon-based NPN junctions, especially when they operate under forward bias conditions. The introduction of defects and traps within the silicon material, as induced by irradiation, fundamentally alters the conductivity of the semiconductor. This alteration stems from a combination of factors. First, the defects act as barriers or recombination sites for charge carriers, impeding their free movement through the crystal lattice. This increased likelihood of carrier trapping and recombination disrupts the smooth flow of charge carriers, diminishing the overall current. Second, the traps introduce energy levels within the semiconductor bandgap, which further affects charge carrier behavior. These energy levels can capture and release charge carriers at different rates, contributing to variations in carrier mobility and recombination processes. As a consequence, the data presented in Figure 2 underscore the pivotal role of irradiation in shaping the performance of silicon-based NPN junctions. These findings carry valuable implications for applications in radiation-sensitive environments and electronics. Understanding the profound impact of irradiation on device characteristics is paramount, as it offers insights into how semiconductor devices respond to ionizing radiation. Such knowledge is invaluable in fields where radiation tolerance and reliability are critical, including aerospace, nuclear technology, and medical equipment. By comprehending the intricate interplay between irradiation and semiconductor behavior, engineers and researchers can design and optimize devices that can withstand or even exploit irradiation in various practical applications, thus ensuring the integrity and functionality of electronic systems in challenging environments.

**Figure 3:**
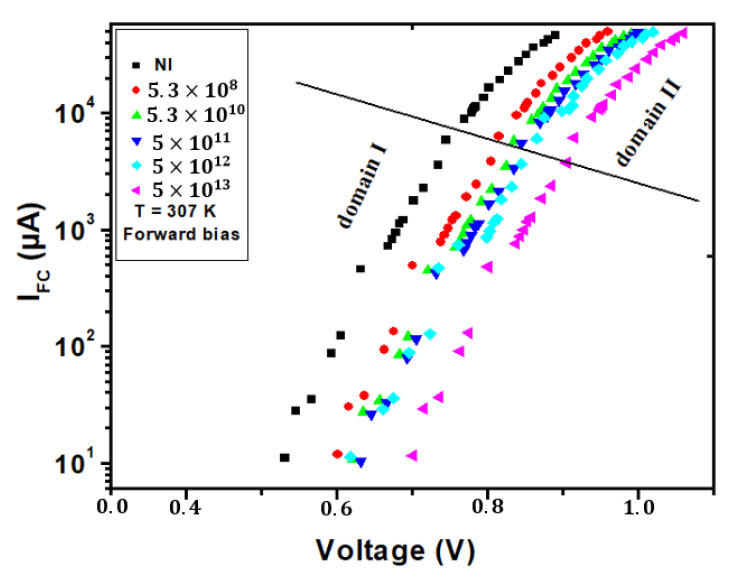
The semilogarithmic representation of the I–V characteristics for bulk silicon NPN emitter-base junctions under forward bias at 307 K is depicted, accounting for the influence of irradiation.

In Figure 3, we delve into the profound influence of irradiation on the I–V (current–voltage) characteristics of a semiconductor device, and the data are ingeniously represented on a semi-logarithmic (semilog) scale at an operating temperature of 307 K. The semilog representation offers a unique perspective that highlights subtle nuances in the I–V curve, which might not be as apparent on a linear scale. It provides a powerful tool for analyzing the behavior of the semiconductor under varying conditions, such as irradiation. One of the most striking revelations in Figure 3 is the division of the I–V curve into two distinct regions, each characterized by different slopes. These two domains suggest that the semiconductor’s behavior is altered significantly by irradiation, resulting in the manifestation of two distinct electrical response modes. The dissimilar slopes indicate a remarkable shift in the device’s electrical characteristics and behavior.

To comprehensively model and make sense of this experimental data, we employ Equation (1) as a mathematical framework. Equation (1) offers insights into the relationship between current (I) and voltage (V) in the context of the irradiated semiconductor device. However, the behavior of the device is not static; it varies with the level of irradiation. To account for these variations, we introduce fitting parameters, denoted as $I_{s},n$, and $R_{s}$, which are unique to each irradiation condition. These parameters allow us to fine-tune the equation for each distinct domain observed in the I–V curve. By extracting and applying these fitting parameters for each irradiation condition and each of the two distinct domains, we gain a nuanced understanding of the behavior of the semiconductor device’s characteristics evolving in response to irradiation. Furthermore, the results of the series resistance ($R_{s}$) and the non-ideality factor (n) are graphically presented in Figure 3. There is a conspicuous elevation in the $R_{s}\;$with increasing irradiation. This phenomenon can be attributed to the incorporation of defects into the microelectronic structure, a phenomenon corroborated by the documented rise in resistivity within the silicon diode, as reported in a prior Ref. [16]. In accordance with earlier observations [27], it has been established that the resistance linearly escalates with increasing irradiation fluences across all cases. These details are critical for both understanding the fundamental physics of the device and for designing semiconductor components that can withstand or even exploit irradiation in various practical applications, such as in radiation-hardened electronics or semiconductor-based radiation sensors.

Calculating the series resistance (*Rs*) in junctionless transistors involves considering the total resistance encountered by electric current within the device. This cumulative resistance arises from various sources, including the semiconductor channel, contact interfaces, and other intermediate materials. It is primarily influenced by physical factors like carrier mobility, impurities, and defects within the semiconductor material. *Rs* includes resistance at the metal-semiconductor contacts and within the conducting channel. Understanding Rs is essential as it directly affects the device’s electrical characteristics, such as the threshold voltage and current–voltage behavior. For comprehensive details on this subject, please refer to the referenced work [28,29,30]. To quantify the experimental damage factor, the outcomes were subjected to fitting. Consequently, the damage factor is defined as follows:

(3) $$R_{s}=R_{s}^{0}(1+k_{R}\emptyset ),$$

where $R_{s}^{0\;}$ and $R_{s}$ are the series resistance before and after irradiation, respectively, $k_{R}\;$is the linear series resistance damage factor, and ∅ is the irradiation fluency.

**Figure 4:**
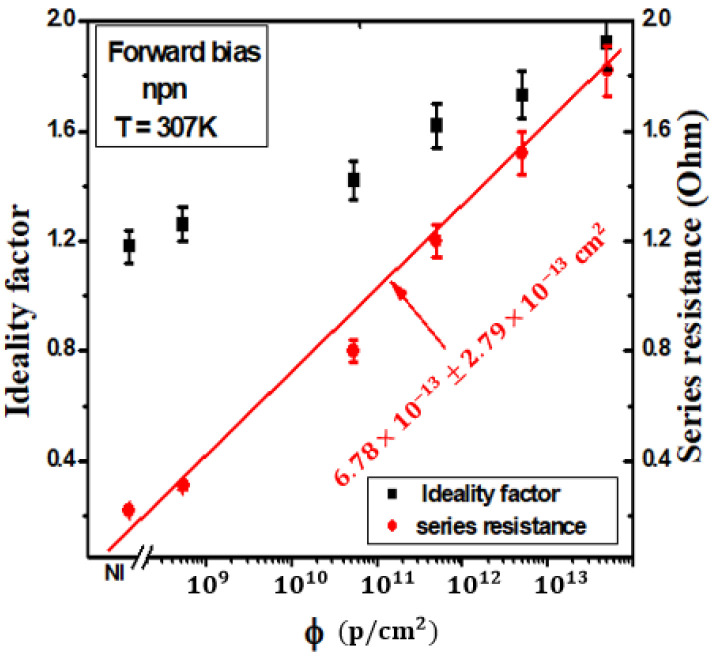
The ideality factor and series resistance of the silicon (npn) emitter-base junction were studied as a function of 1 MeV proton irradiation fluence at 307 K in Domain I. The results in Domain II closely matched those in Domain I. Fitting results are shown by the solid line.

Figure 4 illustrates the investigation of the ideality factor and series resistance of the silicon (NPN) emitter-base junction as they vary with 1 MeV proton irradiation fluence at 307 K within Domain I. The results within Domain II closely align with those observed in Domain I. The fitting results are visually represented by the solid line.

In this Figure, a clear trend emerges as the saturation current significantly increases in response to irradiation. Moreover, this pattern closely mirrors the dependence observed in the series resistance ($R_{s}$), indicating a direct correlation between irradiation levels and both saturation current and series resistance. The experimental data have been subjected to fitting procedures using Equations (3) and (4). Consequently, we derived the linear factor damage coefficients: $k_{R}=(6.87\;\pm \;2.79)\times 10^{-13}\;cm²,\;k_{I}=(2.75\;\pm \;1.12)\times 10^{-12}\;cm²$ (Domain I), and $k_{I}=(1.37\;\pm \;0.69)\times 10^{-12}cm²$ (Domain II). The observed trend highlights that the rate of increase in the non-ideality factor (*n*) is more pronounced than that of the series resistance ($R_{s}$). This outcome implies that the defects introduced due to irradiation, responsible for the elevation in $R_{s}$, do not collectively contribute to the augmentation in n. $k_{I}$ represents the linear saturation current damage factor, signifying the rate at which the saturation current of the semiconductor device increases due to irradiation. In Domain I, $k_{I}$ is higher (2.75 ± 1.12) × $10^{-12}$ cm², indicating a substantial impact on saturation current. In Domain II, $k_{I}$ is lower (1.37 ± 0.69) × $10^{-12}$ cm², reflecting a milder response to irradiation in this domain. These values provide insights into the specific changes in saturation current in response to irradiation within each domain. Overall, Domain I exhibits more substantial changes in saturation current, series resistance, and non-ideality factor in response to irradiation compared to Domain II. These differences indicate that the impact of irradiation on the electrical characteristics of the semiconductor device is more pronounced in Domain I, while Domain II shows a comparatively milder response to irradiation. However, we can establish a saturation current damage factor, analogous to the one defined for $I_{s}$.

(4) $$I_{s}=I_{s}^{0}\left(1+k_{I}\emptyset \right),$$

where $I_{s}^{0}\;$and ${\;I}_{s}$ are the saturation current before and after irradiation, respectively, and $k_{I}\;$is the linear saturation current damage factor.

**Figure 5:**
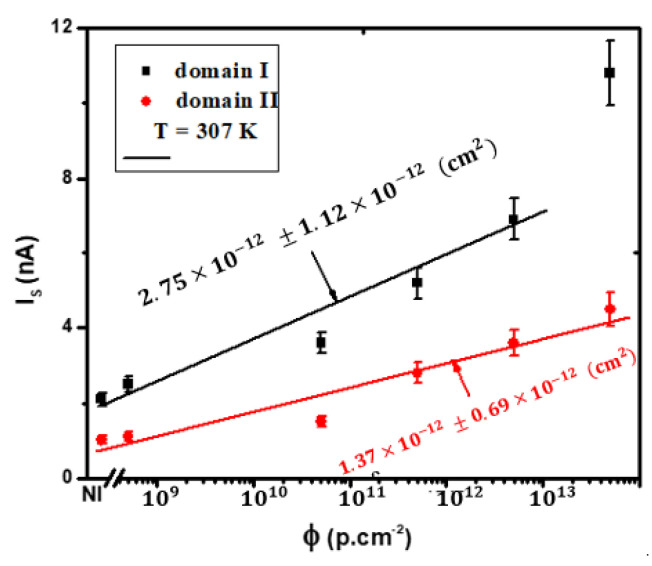
The reverse saturation current of the silicon NPN emitter-base junction at 307 K as a function of irradiation fluences, delineated for two distinct domains. The solid lines represent the outcomes of the fitting procedure.

Figure 5 provides detailed insight into how the reverse saturation current of a silicon NPN emitter-base junction behaves when subjected to varying levels of irradiation at an operating temperature of 307 K. These data are thoughtfully separated into two distinct domains for clarity, and the solid lines on the graph represent the results derived from a fitting procedure. What becomes strikingly evident is the consistent increase in the parameter n as irradiation levels intensify. In specific terms, ‘n’ rises from 1.18 ± 0.06 in the case of unirradiated samples to 1.92±0.07 when the fluence reaches ${5\times 10}^{13}\;cm^{-2}$.

This shift in n is significant because it signifies a change in the predominant mechanisms governing the conduction transport within the semiconductor device; although, for the unirradiated samples, the conduction mechanism at 307 K is primarily dictated by the diffusion component. However, as the irradiation fluence increases, the non-ideality factor n also increases, and this leads to a shift in the conduction mechanism. The device begins to rely more heavily on the generation-recombination component, which involves the creation and recombination of electron–hole pairs, in addition to diffusion. Remarkably, this observed behavior mirrors the changes induced by irradiation in the series resistance ($R_{s}$), as explained earlier. Both the increase in ‘n’ and the elevation in $R_{s}$ suggest that irradiation modifies the underlying physics of the semiconductor, affecting the relative contributions of diffusion and generation-recombination currents to the conduction mechanism. This nuanced understanding of the interplay between n and $R_{s}$ is invaluable in modeling and optimizing semiconductor devices under radiation conditions and is highly relevant in fields where radiation tolerance and performance are critical.

Table 1 serves as a concise and informative representation of irradiation’s impact on the ideality factor n based on observations in Domain II. Interestingly, the behavior of n in response to irradiation closely mirrors the trends observed in Domain I. In both domains, n consistently exceeds the conventional value of 2, prompting intriguing inquiries into the fundamental physics governing the emitter-base junction during irradiation. The observation that n surpasses 2 suggests the possible influence of additional current components beyond those conventionally associated with the ideality factor. This discrepancy implies the existence of further factors or mechanisms within the semiconductor device when exposed to irradiation. As a result, there is a compelling need for comprehensive investigation and analysis to uncover the precise nature of these supplementary components and their impact on the overall junction current. This deeper understanding is crucial for enhancing models and enhancing the reliability and performance of semiconductor devices when operating in radiation-intensive environments.

**Figure 6:**
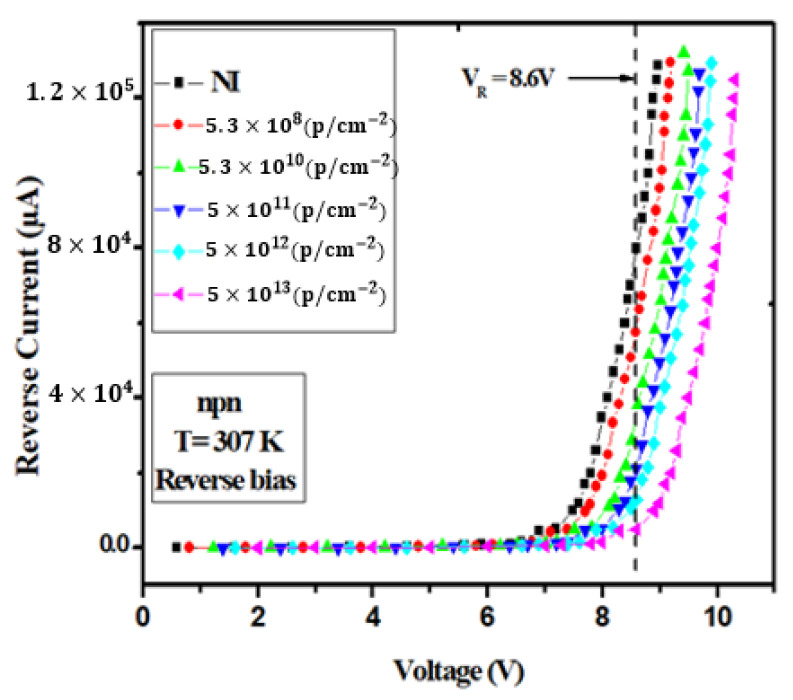
The reverse bias I–V characteristics of silicon (NPN) emitter-base junction at 307 K, considering the influence of proton irradiation. “NI” designates the condition of an unirradiated sample.

Figure 6 displays the reverse bias I–V characteristics of a silicon (NPN) emitter-base junction at 307 K, taking into account the impact of proton irradiation. The label ‘NI’ denotes the condition of an unirradiated sample. The data presented in this figure demonstrate that, for voltages below 7.2 V, the current flowing through the silicon (NPN) emitter-base junction remains practically negligible. However, as the applied voltage exceeds this critical threshold, a remarkable phenomenon unfolds; the current begins to exhibit exponential growth. This remarkable behavior can be attributed to the substantial increase in the electric field intensity within the junction. This intensification of the electric field is sufficient to create electron–hole pairs within the semiconductor material, which, in turn, sets the stage for what is known as the avalanche phenomenon. The avalanche effect occurs when the electric field is strong enough to trigger a chain reaction of electron–hole pair generation. This process significantly amplifies the current, leading to the exponential growth observed in the graph. It is important to note that, as the level of irradiation fluence increases, there is a discernible elevation in the knee voltage, which is the point at which the current starts to exhibit this exponential growth. For instance, in unirradiated samples, the knee voltage measures 7.2 V. However, as the irradiation fluence intensifies to $10^{13}\;p/cm^{2}$, the knee voltage noticeably increases to 8.3 V. Moreover, the observation of higher breakdown voltage in the reverse current at higher radiation levels is indeed an important aspect that warrants clarification. This phenomenon can be attributed to the influence of irradiation on the electrical properties of the semiconductor material. As radiation increases, defects and traps are introduced into the material, affecting its ability to maintain a low reverse current. This correlation between irradiation level and the knee voltage underscores the significant impact of irradiation on the electrical behavior of the junction, providing critical insights for understanding the radiation tolerance and performance that are paramount of the device.

**Figure 7:**
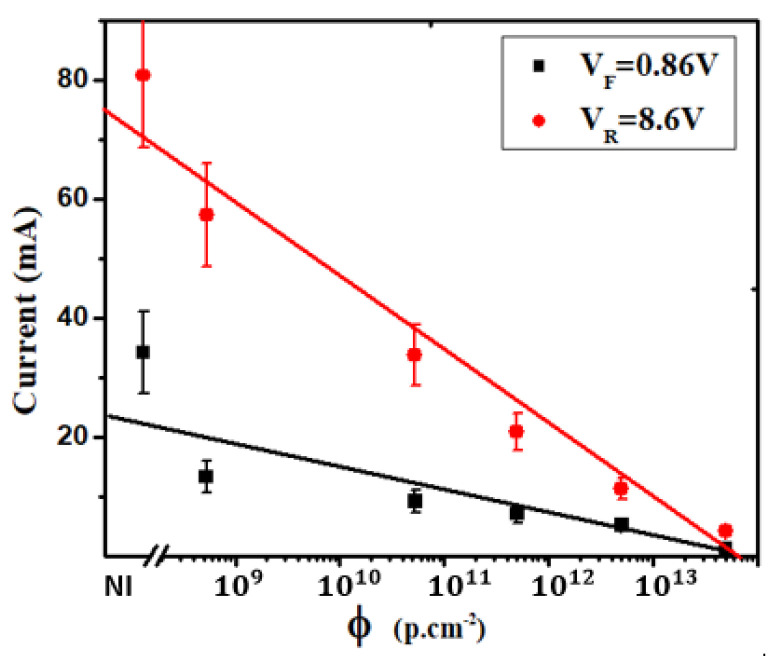
The impact of 1 MeV proton irradiation on the current for constant reverse and forward polarizations at 307 K in silicon NPN emitter-base junction.

Figure 7 depicts the impact of 1 MeV proton irradiation on current at constant reverse and forward polarizations at 307 K in a silicon NPN EBJ. It is evident that the current exhibits a diminishing trend as irradiation fluences increase, a behavior that approximates a linear decline. Consequently, we have defined experimental linear degradation factors, following the same methodology as for $R_{s}$ and $I_{s}$. Specifically, we have determined linear degradation factors of $(6.61\pm 0.98)\times 10^{-16}\;cm^{2}$ per proton for forward bias and $(1.53\pm 0.23)\times 10^{-15}\;cm^{2}$ per proton for reverse bias. It is noteworthy that the degradation of reverse current is more pronounced compared to that of forward current for both polarization states. This observed degradation in both forward and reverse currents can be attributed to the capture of charge carriers by defects introduced into the forbidden band, implying a substantial doping compensation effect induced by irradiation.

It is well-established that the introduction of defects increases as a function of irradiation fluences. These defects manifest at various energy levels within the semiconductor band gap, often linked to different structural defects that can range from specific to complex in nature. These energy levels are established through an experimental technique, including deep-level transient spectroscopy (DLTS). As demonstrated by [16], irradiation in silicon introduces two distinct defects situated at energy levels of 0.19 eV and 0.35 eV, which are attributed to complexes associated with oxygen. Additionally, other defects have been reported, including donor defects at energy levels of 0.24 eV and 0.35 eV, which are ascribed to copper, and acceptor defects at energy levels of 0.49 eV and 0.54 eV, which are linked to gold [31]. Furthermore, it was reported by Watkins et al. [32,33] that this irradiation effect leads to the creation of two distinct defects within the silicon material. The first defect, denoted as the A center, is situated at an energy level of 0.17 eV. This A center is attributed to complexes formed by silicon vacancies and oxygen atoms. The second defect, known as the E center, is located at an energy level of 0.4 eV. It corresponds to complexes formed by silicon vacancies and a weak donor, particularly phosphorus (P). At the temperature of 307 K, the dominant conduction mechanism within Domain I is diffusion. However, with increasing irradiation fluences, the density of generation centers also increases, as discussed earlier. Consequently, conduction becomes increasingly reliant on the generation-recombination component, thereby corroborating the observed rise in the non-ideality factor (n). This observed behavior aligns consistently with the results obtained for $R_{s}$ and $I_{s}$, as per the irradiation dependencies discussed earlier. The factors $k_{R}$ and $k_{I}$ provide additional insights, suggesting that the defects introduced into the silicon junction, which primarily contribute to the increase in $I_{s}$, do not entirely account for the rise in $R_{s}$. Furthermore, the values obtained for n in Domain II suggest that forward current modeling should encompass the consideration of additional components such as tunneling effects, surface diffusion effects, and surface generation-recombination effects. This implies that the surface effects may be attributed to suboptimal passivation of the silicon junctions. For both polarization states, the recurring trend of decreasing current with increasing irradiation fluences is noteworthy. Consequently, we can confidently assert that the principal effect of irradiation is to compensate for doping rather than reinforce it. In other words, the introduction of donor defects is dominated by the introduction of acceptor centers.

## 4. Conclusions

In conclusion, our study investigated the irradiation effects on silicon (NPN) emitter-base junctions at 307 K. We focused on various electrical characteristics, including series resistance ($R_{s}$), saturation current ($I_{s}$), and the non-ideality factor (n), under both forward and reverse polarization. Our findings revealed that irradiation induced significant changes in these parameters, with $R_{s}$ and $I_{s}$ showing linear dependencies on irradiation fluence. Nevertheless, n exhibited a more pronounced increase than $R_{s}$, indicating that defects introduced by irradiation did not contribute equally to both. Moreover, our results demonstrated that irradiation compensated for doping rather than reinforcing it, suggesting a dominant role for acceptor centers. These insights contribute to our understanding of semiconductor behavior under irradiation, with potential implications for the design and reliability of electronic devices in 1 MeV proton-irradiation environments.

## Figures and Tables

**Table 1:** The impact of irradiation on the non-ideality factor (n) of the silicon NPN E-B junction as a function of 1 MeV proton irradiation fluences at 307 K in Domain II, where “NI” designates the condition of no irradiation.

$\Phi \;\left(p.cm^{-2}\right)$	NI	5.3 × 108	5.3 × 1010	5 × 1011	5 × 1012	5 × 1013
n (307 K)	2.1	2.5	2.9	3.4	3.1	3.9

## Data Availability

Not applicable.
